# Prepulse inhibition predicts subjective hearing in rats

**DOI:** 10.1038/s41598-021-98167-6

**Published:** 2021-09-23

**Authors:** Naoki Wake, Kotaro Ishizu, Taiki Abe, Hirokazu Takahashi

**Affiliations:** Department of Mechano-Informatics, Graduate School of Information Science and Technology, The University of Tokyo, 7-3-1 Hongo, Bunkyo-ku, Tokyo, 113-8656 Japan

**Keywords:** Auditory system, Neurological disorders

## Abstract

Auditory studies in animals benefit from quick and accurate audiometry. The auditory brainstem response (ABR) and prepulse inhibition (PPI) have been widely used for hearing assessment in animals, but how well these assessments predict subjective audiometry still remains unclear. Human studies suggest that subjective audiometry is consistent with the ABR-based audiogram, not with the PPI-based audiogram, likely due to top-down processing in the cortex that inhibits PPI. Here, we challenged this view in Wistar rats, as rodents exhibit less complexity of cortical activities and thereby less influence of the cerebral cortex on PPI compared to humans. To test our hypothesis, we investigated whether subjective audiometry correlates with ABR- or PPI-based audiograms across the range of audible frequencies in Wistar rats. The subjective audiogram was obtained through pure-tone audiometry based on operant conditioning. Our results demonstrated that both the ABR-based and PPI-based audiograms significantly correlated to the subjective audiogram. We also found that ASR strength was information-rich, and adequate interpolation of this data offered accurate audiometry. Thus, unlike in humans, PPI could be used to predict subjective audibility in rats.

## Introduction

A quick and accurate assessment of subjective audibility in rodents will substantially benefit the field of auditory research. A variety of protocols have been proposed to test hearing in animals, including behavioral audiograms^1–10^, auditory brainstem response (ABR) (for review^11^), and prepulse inhibition (PPI)^12–18^. A behavioral audiogram based on operant conditioning can be an acceptable benchmark for measuring subjective audibility, comparable to human audiometry^4,6–9^. However, operant conditioning is time-consuming and the training ranges from days to months. Thus, ABR- and PPI-based audiograms are more commonly used to test hearing in animals, as these assessments require only few hours to perform without prior training. However, few studies have quantified the effectiveness and consistency of ABR- and PPI-based audiograms in assessing subjective audibility in animals^19,20^.

Some human studies have shown that subjective audibility is highly correlated with ABR-based audiograms^21,22^, but not with PPI-based audiograms^23–25^. As PPI is derived from the acoustic startle reflex (ASR)—which is influenced by indirect projections from many brain regions (e.g., from the auditory cortex to the caudal pontine reticular nucleus (PnC)^26–29^)—inter-species differences in neural activities that inhibit ASR may cause a discrepancy between subjective audibility and the PPI-based audiogram.

Assuming that the top-down control of the ASR may be qualitatively different in rodents than in humans, the present study challenged the conventional view of discrepancies between subjective audibility and PPI-based audiograms in Wistar rats. PPI-based audiograms are in close agreement with ABR-based audiograms in mice^12^, suggesting that unlike in humans, PPI-based audiograms in Wistar rats predict subjective audibility. However, to the best of our knowledge, there is no direct evidence to show that PPI-based audiograms reliably approximate subjective audiometry in Wistar rats.

The aim of the present study was to evaluate whether and how well ABR- and PPI-based audiograms predict subjective audiometry in rats. To quantify subjective audiometry, we used pure-tone audiometry based on operant conditioning in rats. We then compared subjective audiometry with ABR- and PPI-based audiograms. We demonstrated that the PPI-based audiograms of rats were closely correlated with subjective audibility as well as ABR-based audiograms, supporting the hypothesis that PPI-based hearing tests are reliable in Wistar rats.

## Results

**Figure 1:**
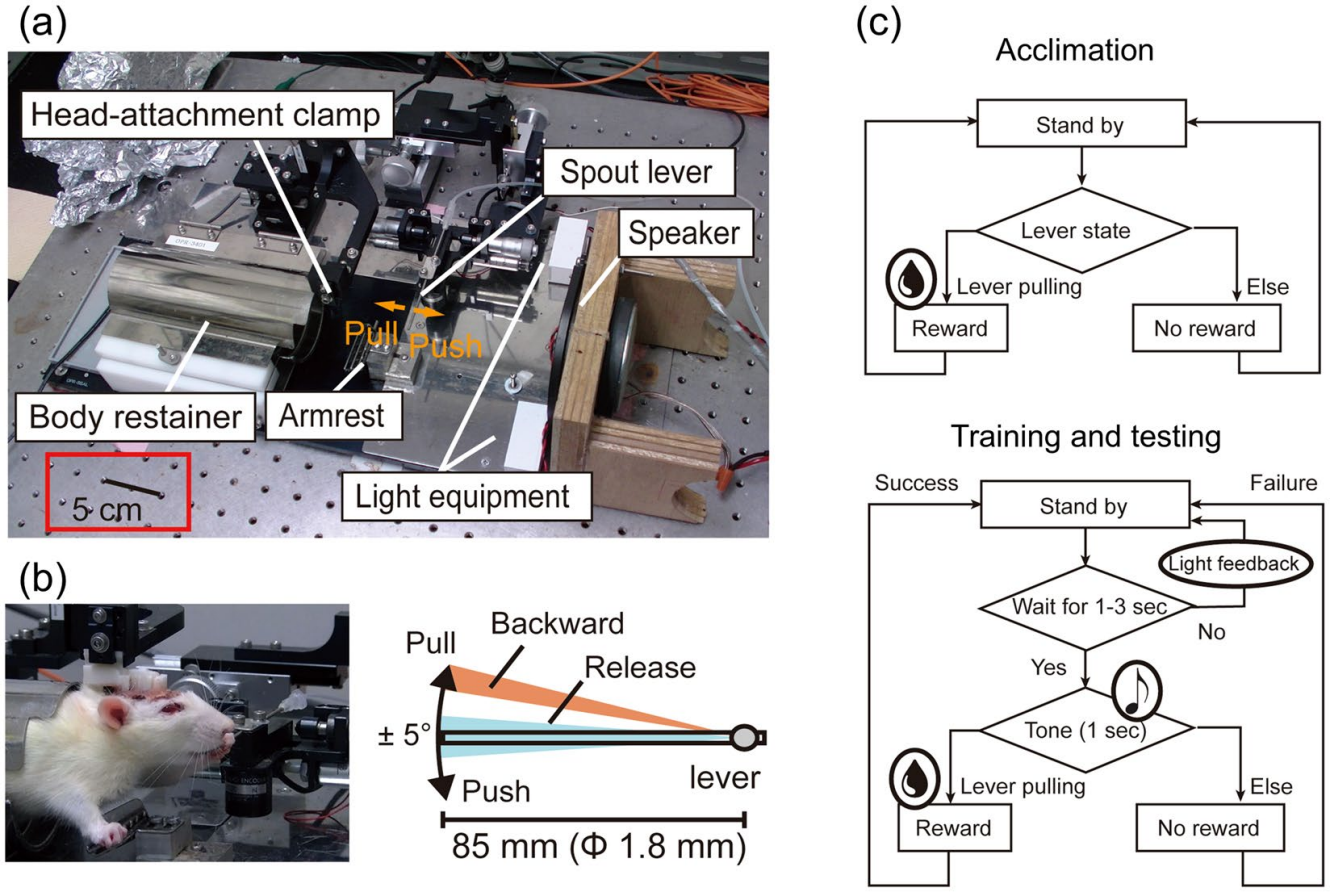
An overview of the experimental system. (**a**) The parts comprising the system. (**b**) A head-restrained animal being trained to pull a spout lever in response to auditory stimuli (see Supplementary Video S1). (**c**) Diagrams of acclimation, training, and testing sessions.

Figure 1a depicts the experimental system that we developed. A head-restrained animal was trained to pull a spout lever in response to auditory stimuli (Fig. 1b). To minimize any pressure on the animal’s head, the animal body was held by a body retainer. Auditory stimuli were presented through a loudspeaker (DLS-108X, Alpine, Tokyo, Japan) placed in front of an animal. The stimuli were calibrated with a 1/4-inch microphone at the pinnas (4939; Brüel & Kjær, Nærum, Denmark). In addition, visual stimuli were provided from LED equipment as feedback signals to promote learning. The sensor and the actuator (OPR-SPL-RM, OPR-7300C) were custom-designed by O'HARA & Co., Ltd., Tokyo, Japan^30^. Auditory stimuli were delivered to the bilateral ears for pure-tone audiometry, ABR, and PPI measurement. All the audiological measurements were carried out in a sound booth, where the background noise level was 32.1 dB (A-weighted equivalent continuous sound level; MT-325, Mothertool Co., Ltd, Nagano, Japan).

Following the acclimation session to the head-restrained condition, the animal was trained to obtain water by pulling the lever only immediately after the auditory stimulus was presented (Fig. 1c). In the main testing session, we tested the animal with tones with varying frequency and sound pressure level (SPL in decibel with respect to 20 μPa) parameters, and estimated the hearing threshold of the tone with a given frequency in a series of 10 assessment sessions. The test SPL was updated according to the performance of the animal in the previous session; the SPL decreased by 5 or 10 dB if ≥ 6 out of the 10 trials were rewarded; otherwise, the SPL increased by 5 dB.

**Figure 2:**
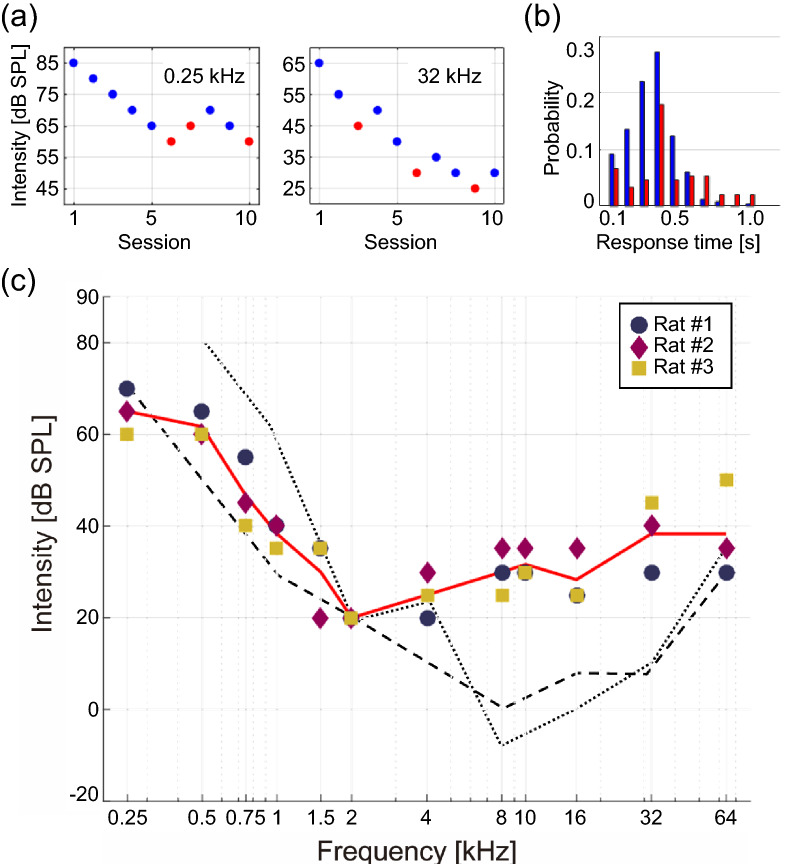
Results of pure-tone audiometry. (**a**) The transitions of test SPL values across sessions for 0.25 and 32 kHz tones. When the subject was rewarded in ≥ 6 out of 10 trials in a given assessment session, the test SPL was lowered in the next assessment session (blue); otherwise, the test SPL was increased in the next session (red). The detection of tone was defined as a success rate of 0.7. (**b**) Probability distribution of reaction times across 4 hearing tests with 4, 8, 16, and 32 kHz tones. The reaction times for successful (blue) and failed (red) trials are shown. Cases when the lever was not pulled into the backward state within 1 s after the sound presentation are not shown. (**c**) Estimated audiograms of animals. A red line indicates the average of all the audiograms. Dashed lines indicate the audiograms of Sprague–Dawley rats^10^ (longer-dashed line) and cotton rats^9^ (shorter-dashed line) reported in previous studies. Both lines were determined by subjective hearing tests.

Figure 2a shows the transitions of test SPL values across sessions for 0.25 and 32 kHz tones. When the subject was rewarded in ≥ 6 out of 10 trials in a given assessment session (blue), the test SPL was lowered in the next assessment session; otherwise, the test SPL was increased in the next session (red). The downward transitions in early sessions indicate that the behavioral responses to tones were reliable. However, when the test SPL was lower than a certain threshold in late sessions, the behavioral responses became unreliable, suggesting that our procedure can define the hearing threshold of the subject.

Figure 2b shows the probability distributions of reaction times in pure-tone audiometry. The blue and red histograms summarize the reaction times for the blue and red assessment sessions, respectively. The reaction times exhibited a monomorphic peak at approximately 0.35 s (blue), whereas those in unsuccessful trials exhibited less clear peaks, suggesting that the behavior of the animal was triggered by sound perception.

Figure 2c shows the audiogram of each animal tested. The hearing thresholds were highest in the low-frequency band (0.25–1.5 kHz), lowest in the mid-frequency band (2–16 kHz), and slightly increased in the high-frequency band (32 and 64 kHz). This trend was similar to that of Sprague–Dawley rats^10^ and cotton rats^9^ reported in previous studies (dashed lines), both of which determined the hearing thresholds of animals by subjective hearing tests.

Table 1 shows the false positive rates in pseudo-trials. False positive rates of 0.2–0.3 guaranteed that random behaviors were very unlikely to achieve the criteria of audibility (a success rate of 0.7; see section “Prepulse inhibition”).

**Table 1:** False positive rates in pseudo-trials.

Frequency (kHz)	0.25	0.5	0.75	1	1.5	2	4	8	10	16	32	64	Mean ± s.d.
Rat#1	0.0	0.3	0.2	0.0	0.0	0.2	0.4	0.1	0.1	0.2	0.4	0.5	0.2 ± 0.2
Rat#2	0.2	0.2	0.2	0.3	0.1	0.2	0.2	0.2	0.0	0.4	0.1	0.2	0.2 ± 0.1
Rat#3	0.4	0.1	0.4	0.1	0.4	0.0	0.4	0.3	0.4	0.4	0.2	0.3	0.3 ± 0.1

**Figure 3:**
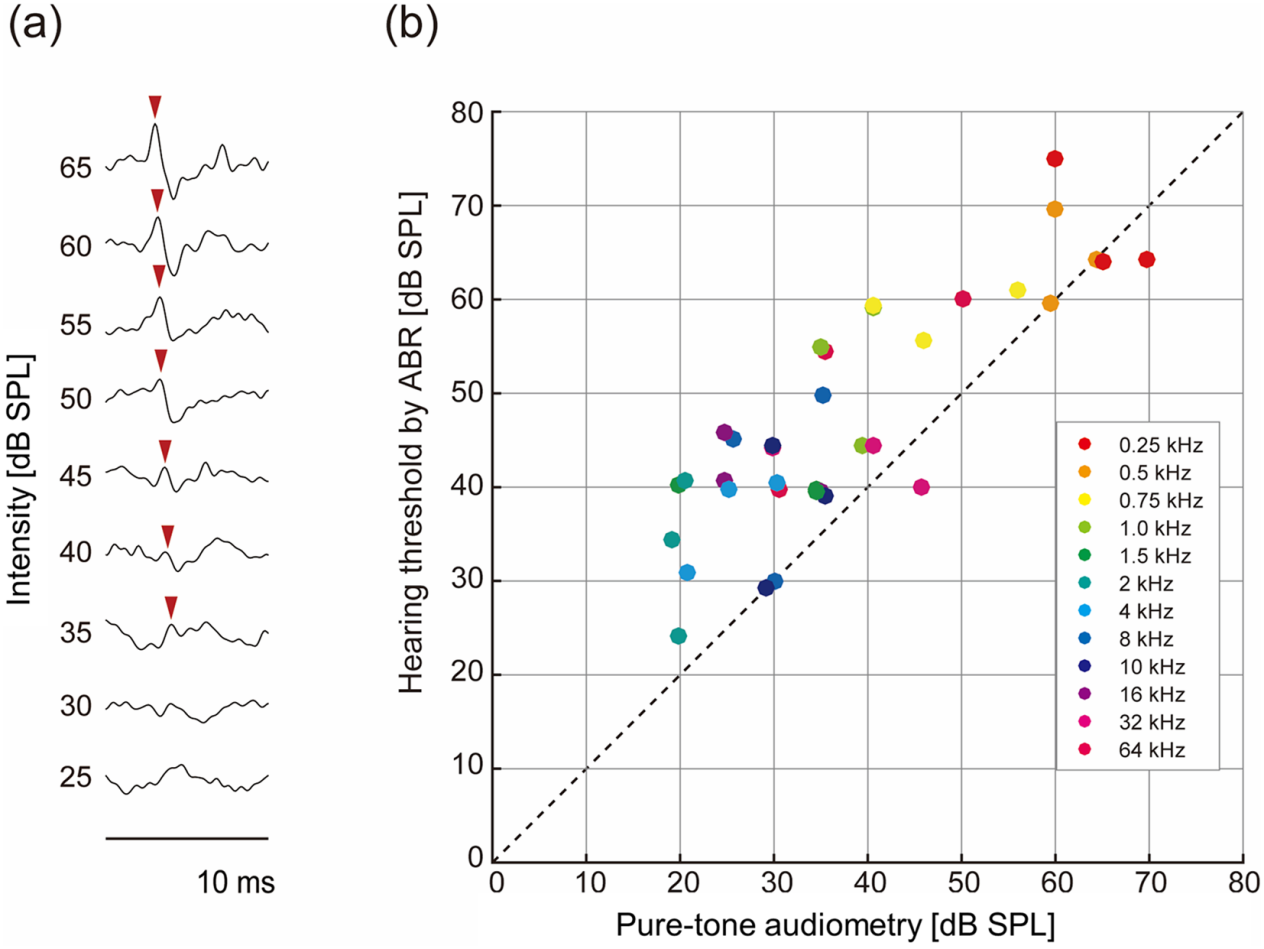
The ABR-based audiogram in relation to the pure-tone audiogram. (**a**) A representative ABR waveform. The peak values (red arrows) were used to determine an ABR threshold. (**b**) The values of the ABR-based audiogram plotted against the values of the pure-tone audiogram.

After a hearing test, we recorded ABR in response to test tones used in the hearing test on the same day. Figure 3a shows representative ABR waveforms. Based on the most distinct wave (red arrows), we visually detected ABR and determined the threshold. The ABR threshold were found to positively correlate with the hearing thresholds in pure-tone audiometry (Fig. 3b; R = 0.84). The correlation was significant for each animal (Rat1: R = 0.94 with *p* = 7.2 × 10^–6^, Rat2: R = 0.84 with *p* = 6.9 × 10^–4^, and Rat3: R = 0.83 with *p* = 8.2 × 10^–4^).

**Figure 4:**
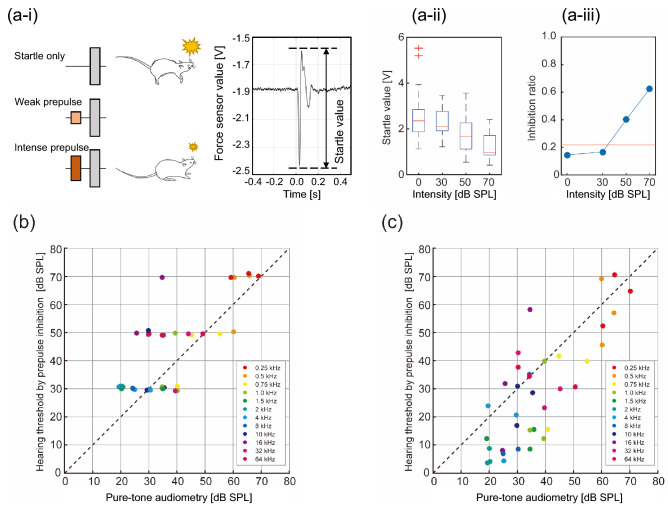
PPI-based audiogram in relation to behavioral audiogram. (**a-i**, left pane) The schematic of stimuli presented for evoking the startle responses. (**a-i**, right pane) Representative raw data of a force sensor output used to determine the strength of the startle response (startle value); (**a-ii**) The boxplots of startle strengths; (**a-iii**) The IR based on the startle strengths. The baseline of IR (red line) is the mean + 1σ of IRs under the startle only conditions across multiple days. The PPI threshold is defined as the smallest SPL where the IR exceeded the baseline. (**b**) The values of the PPI-based audiogram plotted against the values of the behavioral audiogram. (**c**) The values of the PPI-based audiogram after interpolating the IR function, plotted against the values of the pure-tone audiogram.

PPI was investigated as another measure of hearing. In general, a weak pre-stimulus inhibits the startle reflex in response to a preceding post-stimulus, as shown in Fig. 4a-i, left pane. This phenomenon is called PPI, and it is sensitive to the intensity of the pre-stimuli. In this study, startle reflex in response to 95-dB SPL white noise pulse was quantified as a function of the frequency and SPL of the prepulse tone. Figure 4a-i, right pane shows representative raw data of a force sensor output used to determine the strength of the startle response (startle value). Figure 4a-ii,a-iii show boxplots of the startle values and their associated inhibition ratios (IRs—an index of the likelihood of inhibition by a given prepulse; see section “Prepulse inhibition”), respectively, indicating that the PPI of startle responses tended to increase with SPL. The PPI thresholds positively correlated with the hearing thresholds in pure-tone audiometry (Fig. 4b; R = 0.75). The correlation was significant for each animal (Rat1: R = 0.87 with *p* = 2.7 × 10^–4^, Rat2: R = 0.70 with *p* = 1.2 × 10^–2^, and Rat3: R = 0.67 with *p* = 1.8 × 10^–2^). The PPI-based audiogram also positively correlated with the ABR-based audiogram (Supplementary Fig. S1a; R = 0.63). The correlation was significant for one animal (Rat1: R = 0.90 with *p* = 7.8 × 10^−5^, Rat2: R = 0.54 with *p* = 7.2 × 10^−2^, and Rat3: R = 0.56 with *p* = 5.9 × 10^−2^).

The PPI threshold estimates took discrete SPL values (30, 50, and 70 dB) which were too coarse to compare with the hearing thresholds in our pure-tone audiometry that had a 5 dB resolution. Therefore, we redefined the PPI threshold as the SPL, where the baseline IR (red line in Fig. 4a-iii; see section “Prepulse inhibition”) intersected with a line obtained by linear interpolation of the IR, assuming that the startle strength contains richer information about the PPI threshold. This estimate of PPI thresholds exhibited a high correlation with the hearing threshold in pure-tone audiometry (Fig. 4c; R = 0.78). The correlation was significant for each animal (Rat1: R = 0.81 with *p* = 1.4 × 10^–3^, Rat2: R = 0.82 with *p* = 1.1 × 10^–3^, and Rat3: R = 0.79 with *p* = 2.4 × 10^–3^). Similarly, interpolated PPI thresholds were correlated with ABR thresholds (Supplementary Fig. S1b; R = 0.63), where the correlation was significant for two animals (Rat1: R = 0.86 with *p* = 3.3 × 10^−4^, Rat2: R = 0.56 with *p* = 5.9 × 10^−2^, and Rat3: R = 0.71 with *p* = 9.7 × 10^−3^). Thus, our results suggest that discrete values from a PPI test can predict the audiogram of Wistar rats with a fairly fine resolution.

## Discussion

We demonstrated that both ABR- and PPI-based audiograms of rats could predict pure-tone audiograms (Figs. 3b, 4b). As hypothesized, unlike in humans^23–25^, PPI-based audiograms of Wistar rats could predict subjective hearing.

### Pure-tone audiometry

Pure-tone audiometry of Wistar rats exhibited V-shaped audiograms (Fig. 2c), which is consistent with previous reports in cotton rats^9^ and Sprague–Dawley rats^10^. However, the hearing thresholds for 8–32 kHz tones in the present study were higher than those in previous reports. This discrepancy was probably due to differences in tone duration, which was negatively correlated with the detection threshold^31,32^. Tone bursts with 50 ms duration and 1 ms rising/falling were used in the present study, whereas previous studies had used sustained, long-duration tones^9,10^. The short-duration tone had the advantages of reducing variability in reaction times and shortening the total experiment durations, but in turn, resulted in higher hearing thresholds.

In contrast to the detection thresholds for mid-to-high frequency (8–32 kHz) tones, the thresholds for tones below 4 kHz in the present study were similar to those reported previously (Fig. 2c). In this low-frequency range (up to 5 kHz), phase locking to tone stimulus is commonly observed in the cochlear nerves^33^. The resultant periodic firing could be used in temporal coding instead of the rate coding in the cortex^34^. Therefore, detection of low-frequency tones was likely less affected by an accumulation effect during long-lasting tones. Another reason for the robust detection of short low-frequency tones may be the large number of inner hair cells with broad turning properties at low frequencies^35^ that respond to auditory stimuli.

Our experiments showed that the thresholds in the pure-tone audiogram were lower than those in the ABR-based tests (Fig. 3c), as observed in human studies^11^. Several reasons might underlie this discrepancy between these measures of audiometry. First, ABR was characterized through small deflections with amplitudes below 1 µV; this low signal-to-noise (S/N) ratio made automatic ABR detection difficult and visual inspection conservative. Second, the threshold of pure-tone audiometry is subject to the definition of successful tone detection. In our experiments, tone detection was defined as a success rate of 0.7 in a given assessment session. Strict criteria in pure-tone audiometry diminished the discrepancy between these different measures of audiometry. Third, isoflurane anesthesia may have lowered the auditory brainstem sensitivity^36^ compared to ketamine/xylazine anesthesia^12^. Lastly, perceptual learning and cortical plasticity may lower the detection threshold through repeated training of pure-tone detection in pure-tone audiometry^37–40^.

### PPI-based audiometry

Whereas PPI-based audiometry in humans is not significantly correlated with pure-tone audiometry^23–25^, our experiments demonstrated that PPI-based audiograms could predict pure-tone audiograms in Wistar rats (Fig. 4b,c). This discrepancy could be explained by the fact that the top-down modulation of PPI in humans was much stronger than that in Wistar rats. The neuronal circuitry of ASR is modulated by many regions in the brain^26,27^: e.g., the startle was modulated by a peripheral circuits between the auditory nerve fibers, PnC, and the interneurons of the spinal cord^41^, as well as by top-down control from the amygdala, hippocampus, prefrontal cortex, auditory cortex, and many other structures via indirect neural projections to the PnC^27,28,42–44^. Clinical studies have also reported that PPI decreases in patients with schizophrenia^45,46^, thought disorder^47^, and distractibility^48^. Thus, inter-species differences in neural activities that are involved in such top-down modulation of the ASR could explain the extent of the difference between the PPI-based audiogram and the pure-tone audiogram.

While the ABR is widely used to assess animal audibility, it does not always reflect subjective hearing. In practice, ABR is typically recorded in anesthetized animals^12,49^. As suggested by a study of human ABR, the movements of subjects increase the variance of detected ABR peak, causing the uncertain estimation of the signal^50^. On balance, ABR is considered to reflect sensorineural function in the brainstem rather than a subjective perception^51^. In contrast, PPI might be a better predictor of subjective hearing than ABR, as it can be measured only in awake conditions. In fact, noise-induced ABR threshold shifts recover a few months after acoustic trauma^51–53^, whereas PPI is likely to indicate temporary and permanent threshold shifts following noise exposure^12^. As discussed in previous work^12^, the observed PPI results may be attributable to the anatomical fact that the top-down modulation of ASR comes from the auditory structures above the inferior colliculus—i.e., the nuclei upstream of the ABR’s origin. Thus, as occasion demands, PPI could be more suitable than ABR to characterize subjective hearing in Wistar rat experiments.

A common limitation of PPI-based audiometry lies in its difficulty in covering a wide range of test conditions. PPI measurement is generally time-consuming, stemming from a long inter-trial interval that is required to avoid habituation of the ASR, and a number of trials are required to compensate for variability in the ASR; ASR shows variability among trials even when preceding startle sounds were delivered for habituation as a standard PPI protocol (see section “Prepulse inhibition”)^54^. In the present experiments, the inter-trial interval was 20 ± 2 s and each prepulse was tested 30 times; therefore, it took more than 10 min to measure the ASR for each prepulse condition. Furthermore, prolonged experiments are inappropriate for animal welfare. With these constraints, we took 3 days to characterize audiograms with 4 test frequencies and 3 SPLs. The SPL test in this PPI-based audiometry was still discretized with 20 dB SPL intervals, as compared to the 5 dB SPL intervals in pure-tone and ABR-based audiometry.

To overcome this problem, we attempted to use PPI strength instead of merely the PPI threshold, assuming that the startle strength contains more information. Consistent with previous studies^12,13^, PPI in the present work showed stronger inhibition for large SPL values (Fig. 4a-ii,a-iii). Interpolating the IR function converted discretized SPL thresholds in the audiogram into continuous SPL thresholds, which correlated well with the thresholds in the pure-tone audiogram (Fig. 4c). Thus, although the PPI data are discretized and limited, adequate interpolation can offer rapid and accurate audiometry in Wistar rats.

### Methodological consideration

In this study, auditory stimuli were delivered bilaterally under the assumption of symmetric hearing. The subjective audiogram and PPI-base audiogram might be different between symmetric and asymmetric hearing conditions. Particularly in PPI, because of binaural loudness summation, binaural stimuli elicited larger startle responses than monaural stimuli, and in turn, made prepulse inhibition less effective^55^.

Furthermore, a variety of experimental manipulations potentially impact PPI through top-down modulation as PPI is subject to conditions, such as stress levels^56^, social interaction^57^, and drugs^58^. Thus, our results need considerations when extending the results to different experimental conditions.

Lastly, the present study has only investigated the hearing metrics of Wistar rats. As PPI levels differ considerably between rat and mouse strains^59–62^, the finding might not be transferable to rodents in general.

### Conclusion

In conclusion, we demonstrated that, unlike in humans, PPI-based hearing tests in rats predicted the pure-tone audiogram as reliably as ABR-based tests. Thus, our results confirm the implicit assumption in previous studies on Wistar rats, that the inhibition of ASR serves as a measure of the subjective hearing of prepulse tones. We also show that ASR strength is information-rich, and adequate interpolation of this data offers accurate audiometry. While ABR is widely used to measure hearing in animals, PPI could be a promising alternative depending on experimental conditions.

## Materials and methods

This study was conducted in accordance with the National Institutes of Health guide for the care and use of Laboratory animals (NIH Publications No. 8023, revised 1978) and with the recommendations in the ARRIVE guidelines (https://arriveguidelines.org/). Procedures involving the care and use of animals in the present study were approved by the Committee on the Ethics of Animal Experiments at the Research Center for Advanced Science and Technology, The University of Tokyo (RAC170005). All surgeries were performed under isoflurane anesthesia, and every effort was made to minimize suffering. All experiments were carried out in a sound-attenuating chamber (AMC-4015; O’Hara & Co. Ltd., Tokyo, Japan).

### Animal preparation

Three male Wistar rats (300–340 g, 8 weeks old) were used in this study. All the animals were housed singly throughout the experiment. Prior to the experiment, a head fixture was surgically implanted onto the skull under isoflurane anesthesia (Pfizer, Mylan Seiyaku K.K., Tokyo, Japan; 4% for induction and 1–3% for maintenance), which immobilized the head in the system (Fig. 1b). The head fixture was custom-designed and made with a three-dimensional printer (Replicator 2X, Makerbot, NY, USA) with acrylonitrile–butadiene–styrene resin. The design was based on our previous study^63^.

The head of an animal was first fixed to a stereotaxic apparatus (SR-50, Narishige Group, Tokyo, Japan). Lidocaine (0.1–0.3 mL) was injected subcutaneously for local anesthesia. The skin covering the parietal bone was incised, and the muscles were removed to expose the skull. To anchor the head fixture, tiny machine screws (diameter, 1 mm; length, 3 mm; Matsumoto Ind. Co., Ltd., Chiba, Japan) were carefully embedded in five locations on the skull to a depth of 1.2–1.5 mm. Finally, the screw and the head fixture were bonded with dental resin (Super-Bond C & B, Sun Medical, Shiga, Japan; Unifast II, GC Corporation, Tokyo, Japan). A stereotaxic device guided the head fixture to be attached horizontally to the animal’s skull.

After the surgery, the incised wound was protected with an anti-inflammatory ointment (Gentacin, Schering-Plough, Tokyo, Japan). In addition, an analgesic drug (3.3 mg/kg Caspiten, Kissei Pharmaceutical Co., Ltd., Nagano, Japan) and an antibiotic (17 mg/kg Vixirin, Meiji Seika Pharma Co., Ltd., Tokyo, Japan) were intramuscularly injected to minimize pain and suffering of the animals.

### Pure-tone audiometry

Postoperative animals were allowed a recovery period of more than 48 h, during which they were provided with adequate water and food. After full recovery, the animals were water-deprived for 2 days. Throughout the experimental period, the animals were given water occasionally such that their body weight was maintained at ≥ 75% of the initial body weight.

#### Acclimation session

After water deprivation, the animals were trained to acclimate to the head-restrained condition (Fig. 1b), during which they were monitored with an infrared camera placed in the sound-attenuating chamber. Acclimation training was stopped as soon as a rat showed strenuous movements. During the initial training stages, the head-restrained condition may have caused fear or stress in the rats. Therefore, a 10 μL drop of water was given as a reward whenever the animal pulled the lever. The acclimation session lasted for several days until the animal obtained > 500 drops of water in a daily training of 3 h.

#### Training session

In the training session that followed the acclimation session, the animal was trained to obtain water by pulling the lever only immediately after the auditory stimulus was presented (Fig. 1c). The presented sounds were tone bursts of 4, 8, 16, and 32 kHz with a sound pressure of 60 dB SPL and a duration of 50 ms. A stimulus was presented 0.4–4 s after the animal released the lever. Animals were rewarded with a drop of water (10 μL) when they pulled the lever into a backward state, with a rewarding period of 0.1–0.7 s after the tone onset. Up to one drop of water was given per rewarding period. When the lever was pulled outside the rewarding period, no water reward was given and the light was turned on as negative feedback. The training session lasted for several days until the animal was rewarded in 10 successive rewarding periods.

#### Testing session

After the training session, the animal was tested with tones with varying frequency and SPL parameters. The test frequencies were 0.25, 0.5, 0.75, 1, 1.5, 2, 4, 8, 10, 16, 32, and 64 kHz. Previous studies have reported that the hearing threshold of rats is lowest at the mid-frequency tone and highest at low-frequency tone^10,64^. Therefore, the test SPL ranges were selected according to test frequency: 45–85 dB at 0.25 and 0.5 kHz; 5–45 dB at 2, 4, 8, 10, and 16 kHz; and 25–65 dB at 0.75, 1, 1.5, 32, and 64 kHz.

The hearing threshold of the tone with a given frequency was quantified in a series of 10 assessment sessions. In each assessment session, audibility was evaluated for 11 trials presented in a row: 10 trials of an identical tone with a given frequency/SPL condition, and an additional pseudo-trial without any test stimulus or reward. Similar to the training session, lever manipulation within 0.1–0.7 s from the tone onset was associated with a water reward. The pseudo-trial was presented in random order to confirm that the lever manipulation was triggered by test tones, rather than a random strategy.

The first assessment session started with the highest SPL range (i.e., 45, 65, or 85 dB depending on the test frequency). Following every assessment session, the test SPL was updated according to the performance of the animal in the previous session; the SPL decreased by 5 or 10 dB if ≥ 6 out of the 10 trials were rewarded; otherwise, the SPL increased by 5 dB. The sound pressure was decreased by 10 dB when the tested sound pressure was ≥ 15 dB above the lowest test SPL (i.e., 5, 25, or 45 dB depending on the test frequency).

Lever manipulation may depend critically on the animals’ motivation rather than audibility. Therefore, a motivation confirmation session was conducted every 10 assessment sessions, using the largest test SPL. The preceding 10 assessment sessions were included for further analyses only when ≥ 8 out of 10 trials in the following motivation assessment session were rewarded; otherwise, they were discarded. The testing session lasted for several days to test all frequencies.

The hearing threshold of a test frequency was defined as the smallest SPL among the assessment sessions for which an animal was rewarded ≥ 7 out of 10 trials.

### Auditory brainstem response

After a hearing test, we recorded ABR in response to test tones used in the hearing test on the same day. The rats were placed under isoflurane anesthesia (4% for induction and 1% for maintenance), and ABR was recorded from a needle electrode inserted subcutaneously near the apex of the head, with the left auricle as the reference and the right auricle as the ground. The loudspeaker and the head fixation positions were kept identical to those in the hearing test. Tone bursts with a 1 ms rising/falling phase and a 3 ms plateau were presented as test stimuli with an interval of 52.6 ms (i.e., 19 Hz to avoid contamination of the power source noise of 50 Hz). The test SPL ranges were identical to those in the hearing test with 5 dB intervals in descending order. For each test stimulus, neural signals were band-pass filtered at 0.1–3 kHz and recorded at a sampling frequency of 10 kHz, and the data were grand-averaged across 1000 trials. The hearing threshold of ABR was estimated by visual inspection.

### Prepulse inhibition

PPI was investigated as another measure of hearing. Startle reflex in response to white noise (95 dB SPL, 10 ms) was quantified as a function of the frequency (0.25, 0.5, 0.75, 1, 1.5, 2, 4, 8, 10, 16, 32, and 64 kHz) and SPL (30, 50, and 70 dB) of the prepulse tone through a force sensor attached to the floor where the animals were placed. The startle sound was presented every 20 ± 2 s, set randomly between trials. The duration of a prepulse was 50 ms. The interval between the onset of a startle sound and the onset of the prepulse was 100 ms.

In order to avoid a prolonged experiment, 4 frequencies and 3 SPLs were tested in a day. The same 4 frequencies were tested with ABR and Pure-tone audiometry on the same day. To cover all the conditions of test sounds (i.e., 12 frequencies and 3 SPLs), the set of PPI, ABR, and Pure-tone audiometry was measured over 3 days and was completed within 3 weeks.

At the beginning of the daily recording of PPI, the rats were allowed to acclimatize to the experimental environment for 300 s. Next, startle sounds were delivered 10 times for habituation^54,65^. Following the habituation, the daily recording of PPI comprised 30 trials in total. For each test trial, 15 conditions of test sounds were presented in random order: 12 each with predetermined prepulses (i.e., 4 frequencies and 3 SPLs) and 3 conditions without a prepulse (startle only condition).

The startle reflexes were quantified as the peak-to-peak magnitude of the force sensor output from 100 ms before the delivery of the startle sound to 200 ms after. Inhibition of the startle reflex was defined as when the force sensor output was 1σ lower than the mean of the startle only conditions. When the sensor output was below this threshold—i.e., when the animal did not exhibit the distinct amplitude of startle reflex—the trial was labeled as “weak startle due to PPI,” indicating that the animal detected the prepulse. However, because spontaneous movements of animals can hamper the proper detection of startle reflexes in some trials^66^, we discarded trials where the force sensor output exceeded a manually set threshold (0.1–1.6 s prior to the onset of a prepulse).

The inhibition ratio (IR) was defined as the ratio of the “weak startle due to PPI” trials to all the trials. The IR is expected to increase with the prepulse SPL, approaching 1. The baseline IR was also defined as the mean + 1σ of IRs under startle only conditions across multiple days (red line in Fig. 4a-iii). As a measure of the audibility of an animal, we defined the PPI threshold as the smallest SPL where the IR exceeded the baseline (Fig. 4b; either 30, 50, or 70 dB SPL). For obtaining the interpolated PPI thresholds (the ordinate in Fig. 4c), the IRs at different prepulse SPL conditions were first plotted against the SPL, and connected with lines (Fig. 4a-iii, blue line). As this blue line intersected the baseline of IR (red line) in the IR-SPL plane, the interpolated PPI value was defined as the horizontal value of the intersection.

## Supplementary Information

Supplementary Figure S1.

Supplementary Video S1.

## Data Availability

The datasets generated during and/or analysed during the current study are available from the corresponding author on reasonable request.
