# Supplementary material for: Prepulse inhibition predicts subjective hearing in rats
Source: Sci Rep. 2021 Sep 23;11:18902. doi: 10.1038/s41598-021-98167-6 (PMC8460677; doi:10.1038/s41598-021-98167-6)
Supplement: Supplementary file 1 — Supplementary Figure S1. [file 41598_2021_98167_MOESM1_ESM.docx]

Supplementary Information

Title: **Prepulse inhibition predicts subjective hearing in rats**

Authors: **Naoki Wake ^1)^, Kotaro Ishizu ^1)^, Taiki Abe ^1)^, and Hirokazu Takahashi ^1)^**

1) Graduate School of Information Science and Technology, The University of Tokyo, 7-3-1 Hongo, Bunkyo City, Tokyo 113-8656, Japan

Corresponding author:

**Hirokazu Takahashi**

Associate Professor

Department of Mechano-informatics,

Graduate School of Information Science and Technology,

The University of Tokyo

7-3-1 Hongo, Bunkyo-ku, Tokyo, 113-8656, Japan

Phone/Fax: +81-3-5841-0461

Email: takahashi@i.u-tokyo.ac.jp


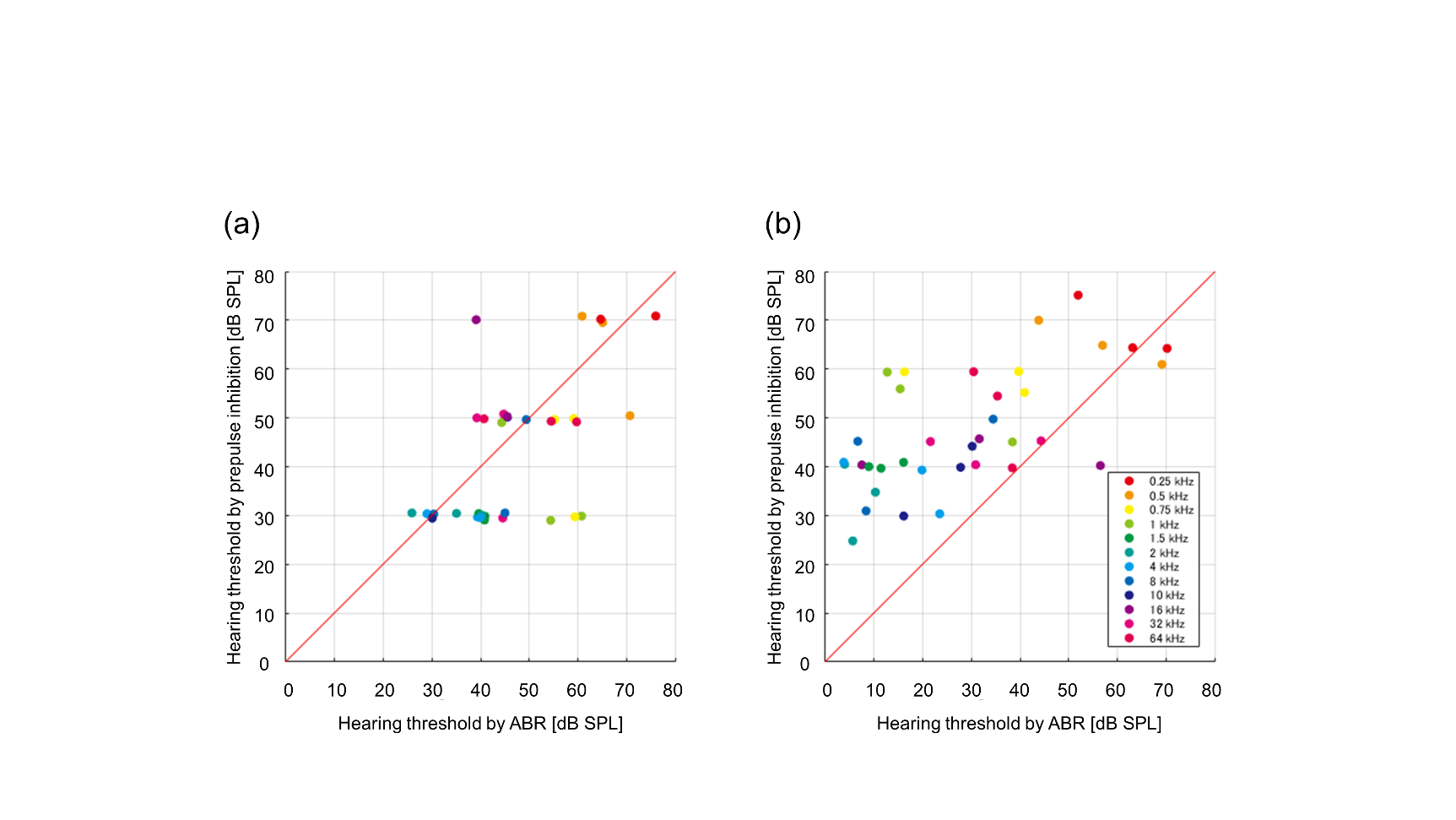
Supplementary Figure S1. PPI-based audiogram in relation to the ABR-based audiogram**:**

**(a):** The values of the PPI-based audiogram plotted against the values of the ABR-based audiogram. **(b):** The values of the PPI-based audiogram after interpolating the IR function, plotted against the values of the ABR-based audiogram.

Supplementary Video S1. Pure-tone audiometry in rats (HearingTest.mp4)
